# Anxiety level modulates endocrine and neuromodulatory responses to maximal exercise and 24-h recovery in elite rowers

**DOI:** 10.3389/fphys.2025.1713588

**Published:** 2025-12-04

**Authors:** Joanna Ostapiuk-Karolczuk, Hanna Dziewiecka, Anna Kasperska, Justyna Cichoń-Woźniak, Małgorzata Reysner, Wojciech Gruszka, Piotr Basta, Sabina Kaczmarczyk, Anna Skarpańska-Stejnborn

**Affiliations:** 1 Department of Biological Sciences, Faculty of Sport Sciences in Gorzów Wielkopolski, University of Physical Education, Poznań, Poland; 2 Department of Palliative Medicine, University of Medical Sciences, Poznań, Poland; 3 Department of Physical Education and Sports, Faculty of Sport Sciences in Gorzów Wielkopolski, University of Physical Education, Poznań, Poland

**Keywords:** anxiety, elite athletes, cortisol, testosterone, serotonin, dopamine, β-endorphin, fatigue

## Abstract

**Introduction:**

Anxiety is a key psychological factor in competitive sport that interacts with physiological stress responses. By modulating neuroendocrine and neurotransmitter activity, it may influence how athletes adapt to maximal effort and recover afterward. The study addressed the gap in understanding how pre-exercise anxiety affects the recovery dynamics of these responses in elite endurance athletes.

**Materials and methods:**

Sixteen highly trained male rowers performed a standardized 2000-m maximal ergometer test and were classified into Low (n = 8) and High anxiety (n = 8) groups based on pre-exercise Sport Competition Anxiety Test (SCAT; low <25, high ≥25) scores using established interpretive guidelines. Venous blood was collected before, immediately after, 1 h, and 24 h post-exercise. Serum cortisol, testosterone, serotonin, dopamine, β-endorphin, anandamide (AEA), and 2-arachidonoylglycerol (2-AG) were analyzed. Testosterone-to-cortisol (T/C) and serotonin-to-dopamine (S/D) ratios were calculated as indices of anabolic-catabolic balance and serotonergic-dopaminergic regulation.

**Results:**

Cortisol increased post-exercise in both groups and remained elevated at 24 h, with prolonged elevation in the High anxiety group (+17.9% vs. +7.8%; *p* = 0.03). Testosterone peaked at 1 h, with a larger rise in the High anxiety group (+42.2% vs. +31.5%; *p* = 0.02). β-endorphin increased post-exercise in both groups (*p* < 0.01). Serotonin remained higher and dopamine recovered more slowly in the High anxiety group (*p* < 0.05). Performance time during the 2000-m test was comparable between groups.

**Conclusion:**

Anxiety level measured before maximal rowing was associated with distinct endocrine and neuromodulatory response patterns, indicating greater internal load despite similar external performance. These findings may support individualized recovery strategies in high-performance sport. The sample size and elite-athlete characteristics may limit the broader applicability of the findings.

## Introduction

In high-performance sports, athletes are simultaneously exposed to systemic load resulting from physical exertion and psychological stress associated with competitive pressure (Armstrong et al., 2022). These stressors do not act in isolation; rather, they accumulate and interact through shared neuroregulatory and autonomic mechanisms, influencing both performance outcomes and recovery dynamics (Tossici et al., 2024). When unmanaged, this cumulative stress load may impair the body’s ability to restore homeostasis after exertion, increasing susceptibility to fatigue, injury, or performance stagnation (Aydemir et al., 2025). Prolonged exposure to overlapping physical and mental stressors has also been implicated in maladaptive responses such as functional overreaching or overtraining syndrome (Armstrong et al., 2022). These risks underscore the need to consider both physical and psychological dimensions when evaluating an athlete’s health and recovery status (Purvis et al., 2010).

These neuroendocrine stress-response pathways are particularly relevant in endurance sports, where sustained high-intensity effort imposes prolonged metabolic, neuromuscular, and psychological load. Among endurance disciplines, competitive rowing combines aerobic and anaerobic output at high intensity, engaging nearly all muscle groups. Elite rowers demonstrate remarkably high maximal oxygen uptake (VO_2_max) approaching 7 L/min. This full-body effort induces extreme physiological stress, severely reducing blood pH and arterial oxygen saturation, while demanding vast pulmonary ventilation (up to 270 L/min) and cardiac output (potentially 40 L/min). Furthermore, maximal effort challenges the central nervous system (CNS), evidenced by a ∼10% decrease in cerebral oxygenation, underscoring the profound systemic demands of this sport (Winkert et al., 2022).

In response to such intense physical exertion, multiple neuroendocrine systems are activated. The hypothalamic-pituitary-adrenal (HPA) axis increases the release of glucocorticoids, such as cortisol, while the hypothalamic-pituitary-gonadal (HPG) axis modulates anabolic hormones, including testosterone (Tossici et al., 2024). Hormonal shifts support adaptation, but if dysregulated, they impair recovery and performance. Specifically, acute, high-intensity exercise typically elicits a significant increase in cortisol level reflecting the body’s immediate stress response and its mobilization of energy substrates to meet the demands of exertion (Hackney and Walz, 2013; Kayacan et al., 2020).

Beyond hormonal regulation, activation of the HPA axis is closely integrated with central neuromodulatory pathways, meaning that stress-related endocrine output and neurotransmitter signaling operate as a coordinated system shaping effort perception, motivation, and recovery. The central nervous system’s response to intense exercise is characterized by changes in neurotransmitter concentrations that modulate effort and contribute to the development of fatigue (Meeu et al., 2021).

Dopamine supports motivation, reward processing, and central drive. Its acute elevation during high-intensity exercise enhances performance capacity, yet sustained or repeated effort can deplete dopaminergic tone, contributing to reduced exercise tolerance and impaired decision-making under fatigue (Basso and Suzuki, 2017). In contrast, serotonin tends to rise progressively during prolonged exertion. Although involved in mood regulation, excessive serotonergic activation, particularly in relation to dopamine, has been linked to central fatigue, reduced arousal, and increased perceived effort, ultimately limiting the ability to sustain maximal output (Cordeiro et al., 2017; Meeusen et al., 2006). In parallel, β-endorphin, released in response to intense physiological stress, functions as an endogenous opioid that reduces pain perception and facilitates continued exertion under high physical load. While beneficial in the short term, an excessive β-endorphin response may mask excessive fatigue, delaying self-regulation and increasing the risk of overexertion or injury (Boecker et al., 2008).

These acute biochemical responses are adaptive; however, inadequate recovery, prolonged psychological stress, or excessive training load can disrupt this balance and impair athletes’ physical and mental health (Meeusen et al., 2013; Kellmann et al., 2018). Understanding these neurochemical dynamics is therefore crucial for optimizing individualized training loads, preventing maladaptive responses, and supporting recovery in high-performance endurance athletes (Halson, 2014).

Several studies indicate that anxiety alone, even in the absence of physical effort, can activate the HPA axis and elevate cortisol and β-endorphin levels before competition, reflecting anticipatory stress responses (van Paridon et al., 2017; Carrasco Páez and Martínez-Díaz, 2021). In elite athletes, heightened trait anxiety has been associated with stronger endocrine reactivity and altered cortisol awakening responses, suggesting reduced adaptive coping capacity under repeated stress (MacDonald and Wetherell, 2019). Moreover, the interpretation of anxiety itself appears relevant, as athletes who appraise anxiety as harmful show stronger cortisol responses than those who view it as facilitative (Turner and Raglin, 1996). Collectively, these findings suggest that psychological readiness may modulate the neuroendocrine response to maximal rowing effort and subsequent recovery, providing the rationale for the present study.

As mentioned above, acute exercise activates the HPA and HPG axes, producing fluctuations in cortisol and testosterone that reflect the balance between catabolic and anabolic processes during and after maximal effort (Cadegiani and Kater, 2017; Hackney, 2020). These endocrine shifts occur alongside changes in serotonin and dopamine, which help regulate motivation and affect during high physiological load (Roelands and Meeusen, 2010). Although these mechanisms are well characterized, much less is known about how pre-competition anxiety influences their recovery dynamics, despite evidence that psychological load can alter stress reactivity and internal load perception (Yang et al., 2024; Li et al., 2025). This dysregulation can impair performance-related behaviors and slow recovery (Basso and Suzuki, 2017).

Against this background, it remains unclear how anxiety shapes the hormonal and neuromodulatory response profile across the mobilization-fatigue-recovery continuum. Clarifying these effects could help guide recovery protocols and psychological strategies for athletes. Understanding these interactions is important for explaining how psychological readiness influences physiological regulation during intense exercise and for developing practical approaches to support performance and athletes' wellbeing.

Although prior studies have examined acute neuroendocrine responses to exercise and the influence of anxiety on stress reactivity, much less is known about how anxiety affects the 24-h recovery dynamics of these systems following maximal effort in elite athletes. This temporal perspective is crucial because recovery processes largely determine training adaptation and perceived internal load.

The present study addresses this gap by combining psychological profiling (SCAT) with time-resolved endocrine and neuromodulatory assessments (pre-exercise, post-exercise, 1 h, and 24 h). This approach provides one of the first characterizations of anxiety-related recovery patterns following maximal rowing effort in elite rowers. We hypothesized that higher pre-competition anxiety would be associated with distinct hormonal and neuromodulatory responses across the 24 h.

Hence, the study aimed to assess whether the anxiety level assessed before a standardized maximal rowing test modulates the acute and 24-h endocrine and neuromodulatory responses in elite rowers. Specifically, we compared changes in cortisol, testosterone, serotonin, dopamine, β-endorphin, anandamide (AEA) and 2-arachidonoylglycerol (2-AG), and the testosterone-to-cortisol (T/C) and serotonin-to-dopamine (S/D) ratios across four time points (pre, immediately post, 1 h, 24 h) between athletes classified as low vs. high anxiety (SCAT <25 vs. ≥ 25).

## Materials and methods

### Ethical approval

The study was approved by the Bioethical Committee of the Poznan University of Medical Sciences (decision no. 685/23, 2023) and conducted in accordance with the Declaration of Helsinki. It was registered on ClinicalTrials.gov (no. NCT06917677). All participants were informed about the study procedures and potential risks and provided written informed consent before participation. Participation was voluntary, and each athlete had the right to withdraw from the study at any time without giving a reason.

### Participants

Sixteen trained male rowers from the Polish Youth National Rowing Team took part in this study. All were medically fit, without any acute or chronic health problems. Inclusion criteria were: at least 5 years of rowing training experience, active membership in the national youth team, completion of a standardized 2000-m ergometer test, and completion of psychological questionnaires assessing anxiety levels. Exclusion criteria included the presence of acute or chronic inflammation, pain or injury, use of anti-inflammatory medications, or non-compliance with the study protocol.

Participants were classified into Low and High Anxiety groups based on their scores on a standardized questionnaire assessing competitive anxiety. This psychological assessment was conducted immediately before the exercise test. Equal group sizes occurred naturally based on the SCAT classification within the national team cohort; no artificial balancing or matching was applied (Table 1).

**TABLE 1:** Participant characteristics, anxiety scores, and 2000 m rowing test outcomes (mean ± SD).

	Low anxiety (n=8)	High anxiety (n=8)	*P* value
SCAT	18.71 ± 1.89	30.71 ± 1.38	**<0.0001**
CSAI-2 Cognitive	1.42 ± 0.42	2.45 ± 0.47	**0.0078**
CSAI-2 Somatic	1.4 ± 0.32	2.02 ± 0.39	**0.0176**
CSAI-2 Confidence	2.75 ± 0.75	2.75 ± 0.62	1.0000
Age (years)	20.75 ± 0.71	20.13 ± 1.12	0.1395
Body mass (kg)	88.53 ± 8.21	87.90 ± 7.29	0.8931
Height (m)	191.13 ± 4.74	190.63 ± 5.02	0.8572
%Fat	11.26 ± 6.19	9.8 ± 3.45	0.8811
Fat free Mass (kg)	81.01 ± 7.04	79.18 ± 5.80	0.6072
Time of exercise (s)	373.7 ± 5.55	373.86 ± 10.69	0.9748
Power (W)	430 ± 19.14	437.36 ± 35.76	0.9825
LA_min_ (nmol/L)	2.14 ± 0.44	2.06 ± 0.73	0.7246
LA_max_ (nmol/L)	10.15 ± 1.28	10.72 ± 2.59	0.9044

SCAT, Sport Competition Anxiety Test; CSAI-2, Competitive State Anxiety Inventory-2 (Cognitive, Somatic, Confidence subscales); LA, blood lactate; LAmin, minimum lactate; LAmax, maximum lactate. Bolded values indicate statistically significant differences between groups (*p* < 0.05).

#### Anthropometric and Body Composition Assessments

Anthropometric measurements, including body mass and composition, were taken before the exercise test using a calibrated scale (Tanita BC-980 MA, Tokyo, Japan) to the nearest 0.05 kg, and body height was measured to the nearest 0.1 cm with a SECA stadiometer (SECA GmbH and Co. KG, Hamburg, Germany) (Table 1).

### Study design

#### Performance test protocol

The rowing performance test was conducted at the beginning of the preparatory phase of the annual training cycle. Following a 5-min individualized warm-up, athletes completed a 2000-m maximal-effort test on a Concept2 rowing ergometer (Concept2 Inc., Morrisville, VT, United States). The 2000-m rowing ergometer test is widely recognized as a valid and competition-relevant performance assessment in elite rowing (Ingham et al., 2002; Cerasola et al., 2020). The ergometer trial served as an official qualifying criterion for the upcoming World Championships. As a decisive selection test, it placed athletes under strong motivational and psychological pressure, like that they experienced before major competitions. This context supported the division into Low and High anxiety groups and allowed analysis of how anxiety influenced physiological responses under competitive conditions.

**FIGURE 1:**
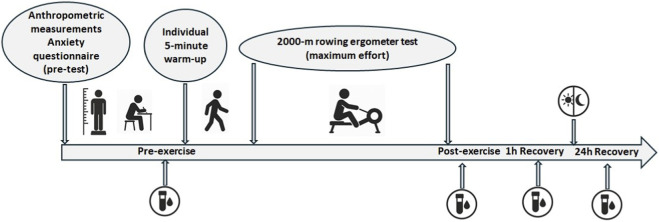
Study design.

Before blood collection, all athletes completed the SCAT (Sport Competition Anxiety Test) and CSAI-2 (Competitive State Anxiety Inventory-2) questionnaires to assess their psychological state. Venous blood samples were collected from the cubital vein at four time points: before the exercise (pre-exercise), immediately after the test (post-exercise), after a 1-h recovery period (1 h Recovery), and 24 h following the test (24 h Recovery) (Figure 1). All testing was performed between 08:00 and 10:00 a.m. to minimize circadian variation in hormonal secretion. Participants refrained from caffeine and alcohol for 24 h before testing. Nutrition, sleep, and daily routines were naturally standardized, as all athletes lived and trained together in the same national team training camp environment.

### Blood analysis

Venous blood samples were collected from the antecubital vein by a trained phlebotomist under standardized conditions at four time points (Pre-exercise, Post-exercise, 1 h Recovery, 24 h Recovery). To minimize the influence of diurnal fluctuations, all blood samples were collected at the same time of day in both groups. The blood was drawn into S-Monovette® serum collection tubes containing a clot activator (Sarstedt AG and Co. KG, Nümbrecht, Germany) using the vacuum system. Following collection, the samples were allowed to clot at room temperature and were subsequently centrifuged at 3000 rpm for 10 min to separate the serum. The serum was aliquoted into microtubes and immediately stored at −80 °C until further biochemical analysis. All procedures were performed in accordance with relevant biosafety standards and under standardized conditions to minimize pre-analytical variability.

Serum concentrations of testosterone and cortisol were measured using enzyme-linked immunosorbent assay (ELISA) kits supplied by DiaMetra (Spello, Italy), according to the manufacturer’s instructions. The analytical sensitivity of the assays was 0.10 ng/mL for testosterone and 2.42 ng/L for cortisol. Serum levels of serotonin, dopamine, β-endorphin (β-EP), anandamide (AEA), and 2-arachidonoylglycerol (2-AG) were determined using ELISA kits from SunRed Biotechnology Company (Shanghai, China), following the manufacturer’s protocols. The detection sensitivities were as follows: serotonin - 0.388 ng/mL, dopamine - 7.043 nmol/L, β-endorphin - 2.451 ng/mL, anandamide - 7.125 ng/mL, and 2-AG - 0.105 ng/L. All analyses were performed using a SPECTROstar Nano microplate reader (BMG Labtech, Germany).

The testosterone-to-cortisol (T/C) and serotonin-to-dopamine (S/D) ratios were calculated to assess anabolic-catabolic and serotonin-dopamine balance.

Lactate concentration (LA) was assessed in capillary blood samples collected immediately after exercise using the Lactat Photometer and a commercially available diagnostic kit (Diaglobal GmbH, Berlin, Germany), according to the manufacturer’s instructions.

### Psychological assessment

To assess the psychological state of the athletes before the exercise test, two standardized questionnaires were administered: the Sport Competition Anxiety Test and the Competitive State Anxiety Inventory-2. Both instruments are widely used in sport psychology to evaluate trait and state anxiety associated with competitive performance. Both measures are widely used to assess trait and state anxiety in competitive contexts, and the Polish adaptations employed in this study have demonstrated satisfactory psychometric properties (Borek-Chudek, 2012; Borek-Chudek, 2019).

The questionnaires were administered in their validated Polish paper-and-pencil versions under standardized conditions, immediately before blood sampling. All participants completed the forms individually in a quiet, controlled environment under the supervision of a trained psychologist.

For group classification, SCAT scores were interpreted according to the original guidelines by Martens et al. (Martens, 1977), distinguishing lower versus higher competitive anxiety. A cut-off score of 25 was applied, reflecting a well-established threshold commonly used in sport psychology research to identify athletes with elevated trait competitive anxiety. Equal group sizes (n = 8 per group) emerged naturally within the national team cohort.

### Statistical analysis

All statistical analyses were performed using *Statistica* 13 (TIBCO Software Inc., Palo Alto, CA, United States), and visualizations were generated in *GraphPad Prism* 8.4.0 (GraphPad Software Inc., La Jolla, CA, United States). Data are presented as mean ± standard deviation (SD), and statistical significance was set at p < 0.05. The normality of data distribution was assessed using the Shapiro-Wilk test. To compare group differences over time and evaluate their interaction, a two-way repeated-measures ANOVA (group × time) was conducted for normally distributed variables. Post-hoc pairwise comparisons were adjusted using the Holm–Bonferroni procedure. Additionally, for each anxiety group (High vs. Low), differences across the four time points (Pre-exercise, Post-exercise, 1 h Recovery, 24 h Recovery) were analyzed using one-way repeated-measures ANOVA for normally distributed variables. Mauchly’s test was applied to verify sphericity; if violated, the Greenhouse–Geisser correction was used. For non-normally distributed data, the Friedman test was used as a non-parametric alternative for within-group comparisons over time, while between-group comparisons at individual time points were evaluated using independent t-tests or Mann-Whitney U tests, as appropriate. Effect sizes were reported alongside p-values to assess the magnitude of observed changes. For ANOVA models, partial eta squared (η^2^_p_) was calculated and interpreted using Cohen’s conventional guidelines: small (≥0.01), medium (≥0.06), and large (≥0.14). For pairwise comparisons, Cohen’s d was used: trivial (<0.2), small (0.2–0.49), moderate (0.5–0.79), and large (≥0.8). Effect size interpretation was emphasized, given the limited sample size (Cohen, 1988). The testosterone-to-cortisol ratio (T/C) and serotonin-to-dopamine (S/D) ratio were calculated as the proportion of total testosterone to total cortisol and total serotonin to total dopamine concentrations, respectively. Relative changes from baseline were expressed as percentage differences calculated as $\left({\text{value}}_{\text{time}}-{\text{value}}_{\text{baseline}}\right)/{\text{value}}_{\text{baseline}}\times 100$ . There was no missing data.

#### Statistical considerations

The number of participants was constrained by the limited availability of elite-level athletes who fulfilled the inclusion criteria. Consequently, *a priori* power analysis (e.g., G*Power) was not feasible. Instead, the study design prioritized the methodological rigor of examining a rare, homogeneous cohort of highly trained athletes subjected to a standardized training regimen. In this context, emphasis was placed on within-subject patterns and effect size interpretation rather than on broad statistical generalizability.

## Results

### Hormonal responses

Both groups demonstrated significant exercise-induced changes in neuroendocrine and neurotransmitter markers, but the magnitude and recovery patterns differed depending on anxiety level.

**FIGURE 2:**
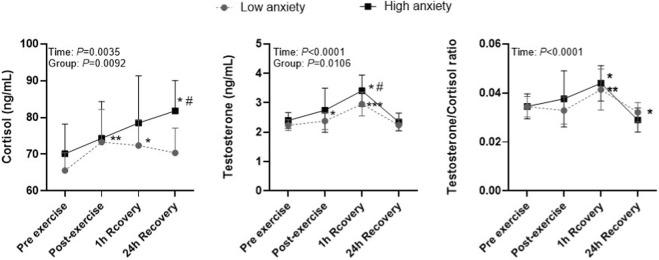
Cortisol, testosterone, and T/C ratio in low- and high-anxiety athletes. Note: Symbols denote statistically significant differences: ^*^ *p* < 0.05, ^**^ *p* < 0.01, ^***^ *p* < 0.001 for changes over time within groups; ^#^ *p* < 0.05, for differences between groups at the same time point.

For cortisol, a significant main effect of time (*p* = 0.0035, η^2^_p_ = 0.21) and group (*p* = 0.0092, η^2^_p_ = 0.14) was found. Cortisol concentrations increased immediately after exercise in both groups, with a large within-group effect in the Low anxiety group (Pre → Post: *d* = 1.03) and a smaller effect in the High anxiety group (*d* = 0.47). At 1 h of recovery, cortisol remained elevated above baseline in both groups (Pre → 1 h: *d* = 1.06 Low; *d* = 0.80 High). After 24 h, cortisol values remained elevated in both groups, with a stronger sustained response in the High anxiety group (Pre → 24 h: *d* = 1.43) compared with the Low anxiety group (*d* = 0.81). At 24 h, cortisol was significantly higher in the High anxiety group compared with the Low anxiety group (*p* < 0.05), indicating a more prolonged stress response. Overall, the High anxiety group showed higher cortisol concentrations across recovery, indicating greater and more prolonged HPA axis activation (Figure 2; Table 2).

**TABLE 2:** Cohen’s d effect sizes for hormonal and neurotransmitter markers.

	Anxiety	Pre vs. Post	Pre vs. 1 h	Pre vs. 24 h	Post vs. 1 h	Post vs. 24 h	1 h vs. 24 h
Cortisol	Low	1.03	1.06	0.81	0.10	0.30	0.23
	High	0.47	0.80	1.43	0.37	0.82	0.31
Testosterone	Low	0.54	2.23	0.05	1.52	0.49	2.14
	High	0.68	2.50	0.18	1.03	0.76	2.54
T/C ratio	Low	0.28	0.02	0.52	0.35	0.16	0.40
	High	0.40	1.64	1.18	0.73	1.12	2.64
Serotonin	Low	0.61	1.63	0.06	1.93	0.63	1.50
	High	1.27	0.29	0.45	0.69	0.72	0.08
Dopamine	Low	3.14	1.62	0.82	0.91	2.06	0.88
	High	2.64	1.09	0.76	0.86	1.50	0.41
S/D ratio	Low	2.04	2.26	0.63	0.07	1.49	1.65
	High	1.86	0.94	0.53	0.33	0.78	0.33
β-EP	Low	2.28	0.48	0.24	1.13	1.17	0.15
	High	1.92	1.87	0.19	0.86	1.74	1.57
AEA	Low	0.12	0.23	0.30	0.12	0.19	0.06
	High	0.09	0.22	0.22	0.04	0.06	0.04
2-AG	Low	0.78	0.91	0.30	0.37	0.42	0.60
	High	0.24	0.28	0.72	0.49	0.51	0.91

Effect size thresholds: trivial (<0.2), small (0.2–0.49), moderate (0.5–0.79), large (≥0.8). T/C, testosterone-to-cortisol ratio; S/D, serotonin-to-dopamine ratio; β-EP, beta-endorphin; AEA, anandamide; 2-AG, 2-arachidonoylglycerol.

For testosterone, a significant main effect of time (*p* < 0.0001, η^2^_p_ = 0.22) and group (*p* = 0.0106, η^2^_p_ = 0.15) was observed. Testosterone concentrations increased significantly post-exercise, reaching peak values at 1 h recovery in both groups, with large effects (Pre → 1 h: *d* = 2.23 in the Low anxiety group; d = 2.50 in the High anxiety group). The relative increase was greater in the High anxiety group (+42.2%) compared with the Low anxiety group (+31.5%). At 1 h, the High anxiety group showed significantly higher testosterone levels than the Low anxiety group (*p* < 0.05). After 24 h, testosterone returned near baseline in the Low anxiety group (Pre → 24 h: *d* = 0.05), whereas in the High anxiety group it declined below baseline (Pre → 24 h: *d* = 0.18), indicating a less favorable anabolic recovery profile in athletes with higher pre-exercise anxiety (Figure 2; Table 2).

For the T/C ratio, a significant main effect of time was detected (*p* < 0.0001, η^2^_p_ = 0.24). The T/C ratio increased significantly after 1 h recovery in both groups with large within-group effects (Pre → 1 h: Low *d* = 1.40; High *d* = 1.64), and the relative elevation was greater in the High anxiety group (+35.6%) compared with the Low anxiety group (+19.6%). At 1 h, the T/C ratio was significantly higher in the High anxiety group than in the Low anxiety group (*p* < 0.05). By 24 h, the T/C ratio declined below baseline in both groups, with large within-group decreases (Pre → 24 h: Low *d* = 1.18; High *d* = 1.12), and the reduction was more pronounced in the High anxiety group (−24.3%) than in the Low anxiety group (−6.6%), resulting in a significantly lower T/C ratio at 24 h in the High anxiety group (*p* < 0.05), indicating a less favorable anabolic-catabolic recovery profile among athletes with higher pre-exercise anxiety (Figure 2; Table 2).

### Neuromodulatory responses

**FIGURE 3:**
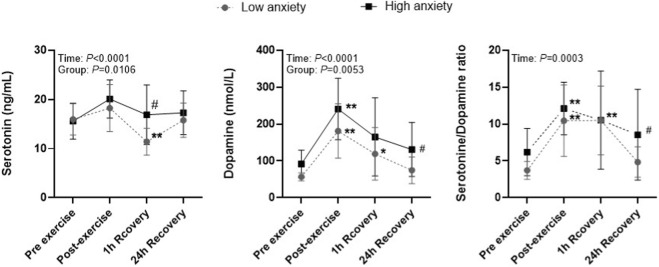
Serotonin, dopamine, and S/D ratio in low- and high-anxiety athletes. Note: Symbols denote statistically significant differences: ^*^ *p* < 0.05, ^**^ *p* < 0.01 for changes over time within groups; ^#^ *p* < 0.05, for differences between groups at the same time point.

For serotonin, a significant main effect of time (*p* < 0.0001,η^2^_p_ = 0.27) and group (*p* = 0.0106, η^2^_p_ = 0.14) was observed. Serotonin levels increased immediately after exercise in both groups (+18.4% Low anxiety; +25.7% High anxiety) with large within-group effects (Pre → Post: *d* = 0.61 Low; *d* = 1.27 High). After 1 h of recovery, serotonin significantly decreased, falling below baseline in the Low anxiety group (−4.8%; Pre → 1 h: *d* = 1.63), whereas in the High anxiety group, the decrease was more gradual and levels remained elevated above baseline (+9.6%; Pre → 1 h: *d* = 0.29). At 24 h, serotonin returned near baseline in the Low anxiety group (Pre → 24 h: *d* = 0.06) and remained slightly elevated in the High anxiety group (Pre → 24 h: *d* = 0.45), indicating more sustained serotonergic activation in athletes with higher pre-exercise anxiety (Figure 3; Table 2).

For dopamine, a significant main effect of time (*p* < 0.0001, η^2^_p_ = 0.29) and group (*p* = 0.0053, η^2^_p_ = 0.16) was observed. Dopamine increased immediately after exercise in both groups, with very large within-group effects (Pre → Post: *d* = 3.14 in the Low anxiety group; *d* = 2.64 in the High anxiety group). At 1 h of recovery, dopamine remained elevated above baseline in both groups (Pre → 1 h: *d* = 1.62 Low; *d* = 1.09 High). At 24 h, dopamine declined toward or slightly below baseline in both groups (Pre → 24 h: *d* = 0.82 Low; *d* = 0.76 High), with slower normalization in the High anxiety group, indicating more prolonged dopaminergic engagement in athletes with higher pre-exercise anxiety (Figure 3; Table 2).

For the serotonin/dopamine ratio, a significant main effect of time (*p* = 0.0003, η^2^_p_ = 0.27) was observed. The S/D ratio increased significantly after exercise, in both groups (Pre → Post: *d* = 2.04 in the Low anxiety group; *d* = 1.86 in the High anxiety group), reflecting acute serotonergic dominance during maximal effort. At 1 h of recovery, the ratio remained elevated, with a larger effect in the Low anxiety group (Pre → 1 h: *d* = 2.26) compared with the High anxiety group (*d* = 0.94). By 24 h, the S/D ratio declined toward baseline in both groups (Pre → 24 h: *d* = 0.63 Low; *d* = 0.53 High). Across all time points, the High anxiety group exhibited a consistently higher S/D ratio (*p* < 0.05), indicating a more pronounced and sustained serotonergic relative to dopaminergic activity pattern during recovery (Figure 3; Table 2).

### Endocannabinoids

**FIGURE 4:**
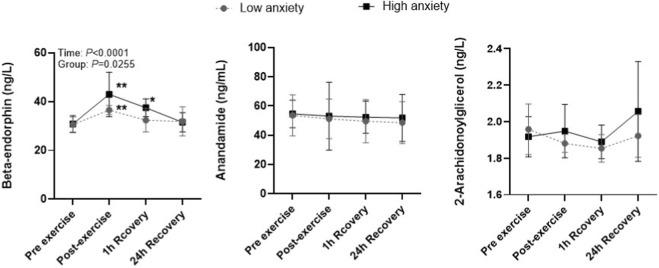
β-endorphin, anandamide, and 2-arachidonoylglycerol in low- and high-anxiety athletes. Note: Symbols denote statistically significant differences: ^*^ *p* < 0.05, ^**^ *p* < 0.01 for changes over time within groups; ^#^ *p* < 0.05, for differences between groups at the same time point.

For β-endorphin, a significant main effect of time (*p* < 0.0001, η^2^_p_ = 0.30) and group (*p* = 0.0255, η^2^_p_ = 0.12) was observed. β-endorphin concentrations increased significantly immediately after exercise in both groups, rising by +50% in the Low anxiety group (Pre → Post: *d* = 2.28) and by +67% in the High anxiety group (*d* = 1.92). At 1 h of recovery, β-endorphin declined but remained above baseline, with a +17% elevation in the Low anxiety group (Pre → 1 h: *d* = 0.48) and a +33% elevation in the High anxiety group (*d* = 1.87). Post-exercise and 1 h values were significantly higher in the High anxiety group compared with the Low anxiety group (*p* < 0.05). By 24 h, β-endorphin returned to baseline in the Low anxiety group (≈0% change, *d* = 0.24), whereas the High anxiety group showed a small residual elevation (+17%, *d* = 0.19), indicating that the opioidergic response resolved within 24 h but was more sustained in athletes with higher anxiety (see Figure 4; Table 2).

For AEA, no significant effects of time, group, or group × time interaction were observed. AEA levels showed only small fluctuations across the measurement time points in both groups. Immediately after exercise, AEA increased by approximately 3%–6% relative to baseline in both the Low anxiety group (Pre → Post: *d* = 0.12) and the High anxiety group (*d* = 0.09). At 1 h, concentrations remained slightly elevated (Pre → 1 h: *d* = 0.23 in the Low anxiety group; *d* = 0.22 in the High anxiety group), and by 24 h they returned to values comparable to baseline (Pre → 24 h: *d* = 0.30 and *d* = 0.22, respectively). These findings indicate that the endocannabinoid response to maximal exercise was small and transient and did not differ between anxiety groups (Figure 4; Table 2).

For 2-AG, no significant effects of time, group, or group × time interaction were observed. 2-AG increased modestly after exercise in both the Low anxiety group and the High anxiety group, corresponding to an approximate +5–12% elevation (Pre → Post: *d* = 0.78 in the Low anxiety group; *d* = 0.24 in the High anxiety group). At 1 h of recovery, 2-AG remained slightly elevated (Pre → 1 h: *d* = 0.91 in the Low anxiety group; *d* = 0.28 in the High anxiety group). By 24 h, 2-AG levels approached baseline in both groups (Pre → 24 h: *d* = 0.30 and *d* = 0.72, respectively). Overall, the endocannabinoid response was small and did not differ between the Low and High anxiety groups (Figure 4; Table 2).

## Discussion

Cortisol reactivity to maximal effort reflected the interaction between physiological and psychological stressors. In the present study, the High anxiety group exhibited greater post-exercise cortisol increases and a delayed return to baseline compared to Low anxiety athletes, consistent with the notion that psychological stress increases HPA stress may augment HPA reactivity in athletes. (MacDonald and Wetherell, 2019). This prolonged recovery pattern suggests enhanced HPA activation in athletes with higher anxiety. Typically, in elite rowers, post-exercise cortisol rises but then returns to baseline (Rämson et al., 2009). However, with repeated maximal tests, cumulative elevations can occur, reflecting competition-related psychophysiological load. Recent frameworks emphasize the interdependence of performance fatigability and perceived fatigability in humans (Behrens et al., 2023). While training usually attenuates the acute response, high life stress may instead prolong elevations and impair recovery.

Sustained elevations of cortisol are also associated with maladaptive training responses (Hackney and Walz, 2013) and may contribute to enduring subjective fatigue; in our previous studies on MMA athletes, significant fatigue persisted despite partial biochemical recovery, including declining cortisol (Ostapiuk-Karolczuk et al., 2025). Beyond acute responses, chronic stress or heightened anxiety can additionally blunt the cortisol awakening response, indicating broader HPA dysregulation that, if recurrent, may promote functional overreaching or overtraining (Aydemir et al., 2025; Purvis et al., 2010).

Testosterone typically rises transiently after high-intensity rowing and returns toward baseline within 24 h (Kraemer and Ratamess, 2005; Jürimäe et al., 2004). In our study, athletes in both groups exhibited a significant post-exercise rise in testosterone with a 1-h peak. However, High-anxiety athletes showed a larger increase followed by a sharper decline at 24 h, whereas Low-anxiety athletes maintained a more stable recovery pattern.

The T/C ratio followed a similar temporal profile, increasing at 1 h and falling below baseline at 24 h in both groups, indicating transient anabolic activation followed by recovery-related suppression. These results align with previous observations of short-lived testosterone elevations following intense effort (Dote-Montero et al., 2021; Ficarra et al., 2024) and extend them by showing that anxiety modulates the magnitude, but not the duration, of this response. Taken together, our data suggest that anxiety amplifies the early androgenic response to maximal effort without altering overall 24-h recovery kinetics, reflecting transient modulation of HPG activity rather than a sustained shift in the anabolic-catabolic equilibrium.

During endurance exercise, serotonin usually increases during and shortly after activity, mainly due to greater availability of tryptophan in the brain and enhanced central serotonin synthesis (Meeu et al., 2021). Zimmer et al. (2016) reported higher serum serotonin following acute intensive aerobic work versus control, and Cordeiro et al. (2017) concluded that elevations, or transient rises near exhaustion, are common, although they show protocol-dependent heterogeneity. Effects on performance are not uniform and depend on intensity, duration, thermal load, and interindividual characteristics (Meeusen et al., 2006; Heijnen et al., 2016).

Dopamine activity, linked to motivation and central drive, rises with increasing task intensity and proximity to exhaustion, often more strongly in well-trained athletes (Basso and Suzuki, 2017; Bracken et al., 2005). However, some protocols report no peripheral changes, highlighting the influence of methodological differences. These findings support central fatigue models emphasizing transmitter balance: a higher serotonin-to-dopamine ratio (S/D) is associated with greater perceived tiredness and reduced arousal, whereas a lower S/D favors sustained effort (Cordeiro et al., 2017; Meeusen et al., 2006).

In the present study, serotonin increased immediately post-exercise and remained elevated at 1 h in both groups, but the subsequent recovery profiles diverged. In the Low anxiety group, serotonin fell rapidly below baseline after 1 h and remained suppressed at 24 h, whereas in the High anxiety group, the decline was more gradual, resulting in comparatively higher serotonergic activity during recovery. Dopamine rose in both groups, peaking at 1 h; by 24 h, normalization was more apparent in Low anxiety athletes, while between-group differences persisted. As a result, the serotonin-to-dopamine ratio (S/D) increased after exercise, peaked at 1 h, and remained elevated at 24 h in the High anxiety group, indicating a slower return to a lower S/D set-point relative to their Low anxiety athletes. This pattern accords with models positing that endurance and high-intensity exercise co-activate serotonergic and dopaminergic systems, with fatigue more accurately indexed by their relative balance than by either transmitter alone (Cordeiro et al., 2017).

Notably, based on anxiety classification, repeated-measures datasets resolving monoaminergic recovery over the first 24 h in elite athletes remain limited; most syntheses emphasize acute responses or pharmacological manipulations, with few studies testing group-by-time effects during recovery (Bracken et al., 2005; Meeusen and Roelands, 2018). The sustained elevation of S/D through 24 h in High anxiety athletes, contrasting with faster normalization and a serotonin decrease in Low anxiety athletes, is notable. Anxiety likely shifts the brain’s short-term serotonin-dopamine balance toward relatively more serotonin. This is consistent with evidence that a higher serotonin-to-dopamine ratio marks central fatigue and slower recovery (Cordeiro et al., 2017; Meeusen et al., 2006; Heijnen et al., 2016). In practice, athletes may feel more tired and less alert in the next training window, which argues for lighter session content, a longer recovery gap, and simple anxiety-reducing strategies (Meeusen et al., 2006; Taylor et al., 2016).

Strenuous endurance and high-intensity efforts elicit β-endorphin (β-EP) elevations, followed by declines during early recovery. Neuroimaging demonstrates central μ-opioid engagement after vigorous endurance running and endogenous opioid release after high-intensity interval training (Boecker et al., 2008; Saanijoki et al., 2018). Recent evidence confirms that β-endorphin release reflects both physical strain and emotional arousal during competition (Marano et al., 2025). At the peripheral level, β-EP consistently rises after demanding endurance or high-intensity bouts, with less reliable changes at lower intensities, indicating an intensity threshold for a pronounced opioidergic response (Heijnen et al., 2016).

In the present study, both anxiety groups exhibited an acute post-exercise increase in β-endorphin. Although concentrations were higher in the High anxiety group immediately after exercise and during early recovery, both groups returned to baseline within 24 h, suggesting that the differences were transient and primarily reflected the acute phase response. These between-group differences occurred despite comparable external performance in the standardized 2,000-m time trial, indicating that anxiety influenced the internal stress response rather than the workload performed.

In athletes, prolonged or highly strenuous tasks induced substantial β-EP responses with slower recovery kinetics (Heijnen et al., 2016). The transient β-endorphin rise observed in our study is consistent with the notion that the opioidergic response mirrors both metabolic load and affective arousal, integrating the physical and emotional aspects of competitive stress. From a practical perspective, a more persistent opioidergic signal in High anxiety athletes may mask early indicators of overload by attenuating perceptions of strain. This may lead to inaccurate assessments of recovery status, whereby athletes or practitioners assume restored readiness despite the persistence of underlying physiological stress, reflecting the hidden costs of fatigue in high-performance sport.

For endocannabinoids, post-exercise anandamide increases have been reported after moderate-to-dynamic running and cycling, and with intense cycling, with intensity-dependent responses peaking at moderate-dynamic ranges (Sparling et al., 2003; Heyman et al., 2012). By contrast, 2-arachidonoylglycerol findings are variable: increases are observed more often after brief resistance tasks, whereas sustained aerobic exercise frequently shows minimal or no 2-AG change (Saanijoki et al., 2018). Methodological factors, including assay approach and sampling timing, critically influence detectable responses, and AEA elevations have been linked to concurrent mood improvements (Hillard, 2018; Crombie et al., 2021).

In the present study, AEA and 2-AG did not change significantly over time in both groups. Although no significant AEA or 2-AG changes were observed, the suggested reduction is inferred from prior studies on stress-induced endocannabinoid modulation, rather than directly demonstrated in our data. Two complementary explanations are supported by prior work: (i) the brief, maximal nature of the ∼6-min 2,000-m effort may lack the duration or intensity that typically elicits AEA elevations during longer continuous aerobic exercise (Sparling et al., 2003; Koltyn et al., 2014); and (ii) heightened activation of the HPA axis and sympathetic nervous system can modulate endocannabinoid signaling. Stress engagement is known to reduce AEA and alter the timing of 2-AG responses, potentially counter-regulating or masking subtle endocannabinoid signals early in recovery (Heyman et al., 2012; Koltyn et al., 2014). Taken together, a pronounced, anxiety-sensitive opioidergic response (β-EP) alongside stable endocannabinoid levels (AEA, 2-AG), helps to explain the more prolonged internal stress signature in the High anxiety group despite equivalent external performance.

## Conclusions

This study demonstrates that pre-competition anxiety modulates neuroendocrine recovery dynamics following maximal rowing effort. Athletes with higher anxiety exhibited prolonged cortisol elevation, stronger β-endorphin responses, and slower serotonergic–dopaminergic normalization, reflecting greater hypothalamic–pituitary axis activation and altered neurochemical recovery kinetics despite comparable physical performance. These findings indicate that competitive anxiety acts as an internal modulator of physiological stress and recovery processes, influencing how athletes restore homeostasis after maximal exertion. Future studies should explore the cumulative effects of psychological stress on endocrine and neuromodulatory regulation in elite sport contexts.

### Practical implications

The present findings highlight that individual differences in competitive anxiety can meaningfully shape the physiological cost of maximal exertion, even when external performance is comparable. In practice, athletes with higher anxiety levels may experience sustained activation of the HPA and opioidergic systems together with a delayed normalization of serotonergic-dopaminergic balance, indicating a greater internal load despite similar training output. Such hidden strain may increase vulnerability to fatigue, impair recovery efficiency, and complicate the interpretation of readiness based solely on performance outcomes. Brief anxiety screening with validated questionnaires could therefore complement traditional monitoring tools, allowing practitioners to identify athletes at higher psychophysiological risk. Integrating psychological profiling with biochemical and neuromuscular markers may support more individualized recovery protocols, reducing the likelihood of overload, guiding training adjustments, and promoting long-term health and performance sustainability in elite sport.

### Study limitations

The present study should be interpreted considering certain constraints. The cohort consisted of a relatively small, homogeneous group of male elite rowers, which provides high experimental control but limits generalization to broader or mixed athletic populations. All athletes were tested while living and training together in the same national team training camp, which ensured naturally standardized nutrition, daily routines, and training environment. Competitive anxiety was assessed as a trait, and future studies incorporating both trait and state dimensions may offer additional insights. Although sampling was carefully standardized, factors such as diurnal variation, nutrition, or sleep could still have contributed to variability. Neuroendocrine markers were determined in peripheral blood, which serves as a practical proxy but does not fully capture central processes. Despite these considerations, the study provides novel evidence linking trait anxiety with distinct short-term endocrine and neuromodulatory responses to maximal rowing.

## Data Availability

The raw data supporting the conclusions of this article will be made available by the authors, without undue reservation.

## References

[B1] Armstrong L. E. Bergeron M. F. Lee E. C. Mershon J. E. Armstrong E. M. (2022). Overtraining syndrome as a complex systems phenomenon. Front. Netw. Physiol. 1, 794392. 10.3389/fnetp.2021.794392 36925581 PMC10013019

[B2] Aydemir M. Makaracı Y. Avcı B. Civil Ürkmez Y. Cintineo H. P. (2025). The psychophysiologic stress and salivary cortisol and alpha-amylase awakening responses to cross-country running competitions in national-level female athletes. J. Strength Cond. Res. 39 (5), e676–e683. 10.1519/JSC.0000000000005055 40030101

[B3] Basso J. C. Suzuki W. A. (2017). The effects of acute exercise on mood, cognition, neurophysiology, and neurochemical pathways: a review. Brain Plast. 2 (2), 127–152. 10.3233/BPL-160040 29765853 PMC5928534

[B4] Behrens M. Gube M. Chaabene H. Prieske O. Zenon A. Broscheid K. C. (2023). Fatigue and human performance: an updated framework. Sports Med. 53 (1), 7–31. 10.1007/s40279-022-01748-2 36258141 PMC9807493

[B5] Boecker H. Sprenger T. Spilker M. E. Bartenstein G. P. Schwaiger M. Hiller R. (2008). The runner's high: opioidergic mechanisms in the human brain. Cereb. Cortex 18 (11), 2523–2531. 10.1093/cercor/bhn013 18296435

[B6] Borek-Chudek D. (2012). Intensywność i ocena lęku sportowego w kontekście poziomu osiągnięć sportowych. Przegl Psychol. 55 (1), 59–77.

[B7] Borek-Chudek D. (2019). Metody do badania cechy lęku w sporcie i emocji we współzawodnictwie sportowym – przykłady polskich adaptacji skal SCAT i CSAI-2R martensa. W: Guszkowska M, gazdowska Z, koperska N (red.). narzędzia pomiaru w psychologii sportu. AWF Józefa Piłsudskiego 5–137.

[B8] Bracken R. M. Linnane D. M. Brooks S. (2005). Alkalosis and the plasma catecholamine response to high-intensity exercise in man. Med. Sci. Sports Exerc 37 (2), 227–233. 10.1249/01.MSS.0000152704.34531.B6 15692317

[B9] Cadegiani F. A. Kater C. E. (2017). Hormonal aspects of overtraining syndrome: a systematic review. BMC Sports Sci. Med. Rehabil. 9, 14. 10.1186/s13102-017-0079-8 28785411 PMC5541747

[B10] Carrasco Páez L. Martínez-Díaz I. C. (2021). Training vs. competition in sport: state anxiety and response of stress hormones in young swimmers. J. Hum. Kinet. 80, 103–112. 10.2478/hukin-2021-0087 34868421 PMC8607774

[B11] Cerasola D. Bellafiore M. Cataldo A. Zangla D. Bianco A. Proia P. (2020). Predicting the 2000-m rowing ergometer performance from anthropometric, maximal oxygen uptake and 60-s mean power variables in national-level young rowers. J. Hum. Kinet. 75, 77–83. 10.2478/hukin-2020-0038 33312296 PMC7706680

[B12] Cohen J. (1988). Statistical power analysis for the behavioral sciences. 2nd ed. Hillsdale (NJ): Lawrence Erlbaum Associates.

[B13] Cordeiro L. M. S. Rabelo P. C. R. Moraes M. M. Teixeira-Coelho F. Coimbra C. C. Wanner S. P. (2017). Physical exercise-induced fatigue: the role of serotonergic and dopaminergic systems. Braz J. Med. Biol. Res. 50 (12), e6432. 10.1590/1414-431X20176432 29069229 PMC5649871

[B14] Crombie K. M. Sartin-Tarm A. Sellnow K. Ahrenholtz R. Lee S. Matalamaki M. (2021). Exercise-induced increases in anandamide and BDNF during extinction consolidation contribute to reduced threat following reinstatement: preliminary evidence from a randomized controlled trial. Psychoneuroendocrinology 132, 105355. 10.1016/j.psyneuen.2021.105355 34280820 PMC8487992

[B15] Dote-Montero M. Martínez-Alemán S. R. Amaro-Gahete F. J. Acosta F. M. Migueles J. H. Mora-González J. (2021). High-intensity interval training improves cardiorespiratory fitness and hormonal responses in healthy young men: the cross-over FITHIIT study. Scand. J. Med. Sci. Sports 31 (4), 835–844. 10.1111/sms.13999

[B16] Ficarra G. Rottura M. Mannucci C. Caccamo D. Bitto A. Trimarchi F. (2024). Testosterone/cortisol ratio: gender effect and prediction of podium results in beach sprint master rowers. Front. Sports Act. Living 6, 1466619. 10.3389/fspor.2024.1466619 39687495 PMC11646765

[B17] Hackney A. C. (2020). Hypogonadism in exercising males: dysfunction or adaptive-regulatory adjustment? Front. Endocrinol. (Lausanne) 11, 11. 10.3389/fendo.2020.00011 32082255 PMC7005256

[B18] Hackney A. C. Walz E. A. (2013). Hormonal adaptation and the stress of exercise training: the role of glucocorticoids. Trends Sport Sci. 20 (4), 165–171. 29882537 PMC5988244

[B19] Halson S. L. (2014). Monitoring training load to understand fatigue in athletes. Sports Med. 44 (Suppl. 2), S139–S147. 10.1007/s40279-014-0253-z 25200666 PMC4213373

[B20] Heijnen S. Hommel B. Kibele A. Colzato L. S. (2016). Neuromodulation of aerobic exercise-A review. Front. Psychol. 6, 1890. 10.3389/fpsyg.2015.01890 26779053 PMC4703784

[B21] Heyman E. Gamelin F. X. Goekint M. Piscitelli F. Roelands B. Leclair E. (2012). Intense exercise increases circulating endocannabinoid and BDNF levels in humans-possible implications for reward and depression. Psychoneuroendocrinology 37 (6), 844–851. 10.1016/j.psyneuen.2011.09.017 22029953

[B22] Hillard C. J. (2018). Circulating endocannabinoids: from whence do they come and where are they going? Neuropsychopharmacology 43 (1), 155–172. 10.1038/npp.2017.130 28653665 PMC5719092

[B23] Ingham S. A. Whyte G. P. Jones K. Nevill A. M. (2002). Determinants of 2,000 m rowing ergometer performance in elite rowers. Eur. J. Appl. Physiol. 88 (3), 243–246. 10.1007/s00421-002-0699-9 12458367

[B24] Jürimäe J. Mäestu J. Purge P. Jürimäe T. (2004). Changes in stress and recovery after heavy training in rowers. J. Sci. Med. Sport 7 (3), 335–339. 10.1016/S1440-2440(04)80028-8 15518298

[B25] Kayacan Y. Makaracı Y. Ozgocer T. Ucar C. Yıldız S. (2020). Cortisol awakening response and heart rate variability in the menstrual cycle of sportswomen. Res. Q. Exerc Sport 92 (4), 760–769. 10.1080/02701367.2020.1774486 32853053

[B26] Kellmann M. Bertollo M. Bosquet L. Brink M. Coutts A. J. Duffield R. (2018). Recovery and performance in sport: consensus statement. Int. J. Sports Physiol. Perform. 13 (2), 240–245. 10.1123/ijspp.2017-0759 29345524

[B27] Koltyn K. F. Brellenthin A. G. Cook D. B. Sehgal N. Hillard C. (2014). Mechanisms of exercise-induced hypoalgesia. J. Pain 15 (12), 1294–1304. 10.1016/j.jpain.2014.09.006 25261342 PMC4302052

[B28] Kraemer W. J. Ratamess N. A. (2005). Hormonal responses and adaptations to resistance exercise and training. Sports Med. 35 (4), 339–361. 10.2165/00007256-200535040-00004 15831061

[B29] Li Y. Ren Y. Du Z. Li M. Jiang J. (2025). Competitive pressure, psychological resilience, and coping strategies in athletes’ pre-competition anxiety. Sci. Rep. 15, 35467. 10.1038/s41598-025-19213-1 41073549 PMC12513990

[B30] MacDonald D. Wetherell M. A. (2019). Competition stress leads to a blunting of the cortisol awakening response in elite rowers. Front. Psychol. 10, 1684. 10.3389/fpsyg.2019.01684 31379693 PMC6657667

[B31] Marano L. Tommasini E. Missaglia S. Vago P. Rampinini E. Bosio A. (2025). Influence of age and fitness level on β-endorphin response to acute aerobic exercise in healthy men. Sport Sci. Health 21, 1513–1520. 10.1007/s11332-025-01367-0

[B32] Martens R. (1977). Sport competition anxiety test. Champaign, IL: Human Kinetics.

[B33] Meeusen R. Van Cutsem J. Roelands B. (2021). Endurance exercise-induced and mental fatigue and the brain. Exp. Physiol. 106 (12), 2294–2298. 10.1113/EP088186 32176398

[B34] Meeusen R. Roelands B. (2018). Fatigue: is it all neurochemistry? Eur. J. Sport Sci. 18 (1), 37–46. 10.1080/17461391.2017.1296890 28317427

[B35] Meeusen R. Watson P. Hasegawa H. Roelands B. Piacentini M. F. (2006). Central fatigue: the serotonin hypothesis and beyond. Sports Med. 36 (10), 881–909. 10.2165/00007256-200636100-00006 17004850

[B36] Meeusen R. Duclos M. Foster C. Fry A. Gleeson M. Nieman D. (2013). Prevention, diagnosis, and treatment of the overtraining syndrome: joint consensus statement of the european college of sport science and the American college of sports medicine. Med. Sci. Sports Exerc. 45 (1), 186–205. 10.1249/MSS.0b013e318279a10a 23247672

[B37] Ostapiuk-Karolczuk J. Dziewiecka H. Bojsa P. Cieślicka M. Zawadka-Kunikowska M. Wojciech K. (2025). Biochemical and psychological markers of fatigue and recovery in mixed martial arts athletes during strength and conditioning training. Sci. Rep. 15 (1), 24234. 10.1038/s41598-025-09719-z 40624253 PMC12234679

[B38] Purvis D. Gonsalves S. Deuster P. A. (2010). Physiological and psychological fatigue in extreme conditions: overtraining and elite athletes. PMR. 2 (5), 442–450. 10.1016/j.pmrj.2010.03.025 20656626

[B39] Rämson R. Jürimäe J. Jürimäe T. Mäestu J. (2009). Behavior of testosterone and cortisol during an intensity-controlled high-volume training period measured by a training task-specific test in men rowers. J. Strength Cond. Res. 23 (2), 645–651. 10.1519/JSC.0b013e318196b801 19197202

[B40] Roelands B. Meeusen R. (2010). Alterations in central fatigue by pharmacological manipulations of neurotransmitters in normal and high ambient temperature. Sports Med. 40 (3), 229–246. 10.2165/11533670-000000000-00000 20199121

[B41] Saanijoki T. Tuominen L. Tuulari J. J. Nummenmaa L. Arponen E. Kalliokoski K. (2018). Opioid release after high-intensity interval training in healthy human subjects. Neuropsychopharmacology 43 (2), 246–254. 10.1038/npp.2017.148 28722022 PMC5729560

[B42] Sparling P. B. Giuffrida A. Piomelli D. Rosskopf L. Dietrich A. (2003). Exercise activates the endocannabinoid system. Neuroreport 14 (17), 2209–2211. 10.1097/00001756-200312020-00015 14625449

[B43] Taylor J. L. Amann M. Duchateau J. Meeusen R. Rice C. L. (2016). Neural contributions to muscle fatigue: from the brain to the muscle and back again. Med. Sci. Sports Exerc 48 (11), 2294–2306. 10.1249/MSS.0000000000000923 27003703 PMC5033663

[B44] Tossici G. Zurloni V. Nitri A. (2024). Stress and sport performance: a PNEI multidisciplinary approach. Front. Psychol. 15, 1358771. 10.3389/fpsyg.2024.1358771 38495423 PMC10940545

[B45] Turner P. E. Raglin J. S. (1996). Variability in precompetition anxiety and performance in college track and field athletes. Med. Sci. Sports Exerc. 28 (3), 378–385. 10.1097/00005768-199603000-00014 8776227

[B46] van Paridon K. N. Timmis M. A. Nevison C. M. Bristow M. (2017). The anticipatory stress response to sport competition; a systematic review with meta-analysis of cortisol reactivity. BMJ Open Sport Exerc. Med. 3 (1), e000261. 10.1136/bmjsem-2017-000261 29177073 PMC5604718

[B47] Winkert K. Steinacker J. M. Koehler K. Treff G. (2022). High energetic demand of elite rowing: implications for training and nutrition. Front. Physiol. 13, 829757. 10.3389/fphys.2022.829757 35514350 PMC9062098

[B48] Yang L. Zhang Z. Zhang J. Veloo A. (2024). The relationship between competitive anxiety and athlete burnout in college athlete: the mediating roles of competence and autonomy: [author]. BMC Psychol. 12 (1), 396. 10.1186/s40359-024-01888-2 39020424 PMC11256448

[B49] Zimmer P. Stritt C. Bloch W. Schmidt F. P. Hübner S. T. Binnebößel S. (2016). The effects of different aerobic exercise intensities on serum serotonin concentrations and their association with stroop task performance: a randomized controlled trial. Eur. J. Appl. Physiol. 116 (10), 2025–2034. 10.1007/s00421-016-3456-1 27562067

